# Acute Limb Ischemia in an Infant Following Inadvertent Femoral Arterial Injury During Central Venous Access

**DOI:** 10.1002/ccr3.73073

**Published:** 2026-07-05

**Authors:** Anas El Hanbali, Hicham Ziani, Leila Drouzi, Hamza Talbi, Zakariya Hamdani, Mahmoud Byadi, Meryem Ennafiri, Noussaiba Nabil, Larbi Ed‐Dafali, Saad El Harrak, Aziza Bentalha, Alae Koraichi, Salma Ech Echerif El Kettani

**Affiliations:** ^1^ Emergency Department, Children's Hospital, Ibn Sina University Hospital Center Faculty of Medicine and Pharmacy of Rabat, Mohammed V University Rabat Morocco; ^2^ Pediatric Multidisciplinary Intensive Care Unit, Children's Hospital, Ibn Sina University Hospital Center Faculty of Medicine and Pharmacy of Rabat, Mohammed V University Rabat Morocco

**Keywords:** acute limb ischemia, central venous catheterization, femoral artery thrombosis, iatrogenic vascular injury, pediatric vascular access, ultrasound guidance

## Abstract

Acute limb ischemia is an uncommon but potentially limb‐threatening complication in pediatric patients. Arterial thrombosis secondary to iatrogenic vascular trauma during vascular access procedures in children has been described in the literature, most frequently following catheter‐based interventions involving the femoral artery. In contrast, cases arising after inadvertent arterial puncture during femoral venous catheterization are only rarely reported. We describe the case of a 13‐month‐old child who developed acute ischemia of the right lower limb following accidental femoral artery puncture during a single attempt at femoral central venous catheterization using the landmark technique, performed by a senior operator experienced in pediatric vascular access. A 4‐Fr catheter was inserted via the Seldinger technique. Despite initially reassuring procedural indicators and prompt manual compression after the error was recognized, the patient developed clinical signs of acute arterial occlusion. Imaging confirmed the diagnosis, and emergency thrombectomy was successfully performed, resulting in a favorable outcome. This case is particularly instructive because it demonstrates that severe acute limb ischemia may occur after a single landmark‐guided femoral venous puncture, even when performed by an experienced operator. It highlights the importance of routine ultrasound guidance for central venous access in pediatric patients, and the need for careful postoperative clinical monitoring to enable early recognition and prompt management of such complications.

## Key Clinical Message

Acute limb ischemia may occur after inadvertent femoral arterial injury during pediatric central venous catheterization, even after a single puncture attempt by an experienced operator. Early recognition, urgent vascular intervention, and routine ultrasound guidance are key to preventing permanent sequelae.

## Graphical abstract

Clinical image showing cyanosis of the right lower limb consistent with acute limb ischemia.

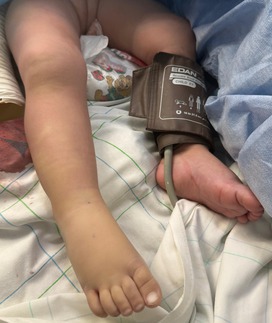

## 1 Introduction

Vascular access is a fundamental component of diagnostic and therapeutic management in pediatric patients, particularly in emergency and critical care settings. Nevertheless, it remains challenging due to anatomical and physiological specificities that increase the risk of complications compared with adults.

Iatrogenic vascular injuries are influenced by several factors, including access site, puncture technique, anatomical proximity of venous and arterial structures, and the small caliber of vessels. The landmark‐based technique has been associated with an increased risk of inadvertent arterial puncture, particularly at the femoral site where the vein and artery lie in close apposition.

The novelty of this case lies in the occurrence of severe acute limb ischemia after a single, uncomplicated attempt at landmark‐guided femoral venous catheterization in an infant, without multiple punctures or obvious technical difficulty, and despite initially reassuring intra‐procedural findings. This underscores that life‐threatening arterial injury may develop even under seemingly favorable conditions and reinforces the critical importance of ultrasound guidance and heightened postoperative vigilance in pediatric vascular access.

## 2 Case History

A 13‐month‐old infant was admitted for surgical management of a left supracondylar humeral fracture. The patient had no significant medical or surgical history.

Preoperative assessment revealed a child in good general condition, awake and responsive, afebrile, and classified as ASA physical status I. Hemodynamics were stable (blood pressure 80/50 mmHg, heart rate 103 bpm). The extremities were warm and well perfused, with symmetrical, palpable peripheral pulses, and no cardiac murmurs. Peripheral venous access was considered poor. The patient weighed 10 kg (+0.8 SD).

After transfer to the operating room, standard monitoring was applied and anesthetic induction was initiated with sevoflurane via face mask. Intravenous anesthesia was then administered using fentanyl and propofol, and a laryngeal mask airway was placed without difficulty.

Shortly after induction, the patient developed significant bradycardia associated with circulatory instability. Intravenous atropine boluses resulted in only partial improvement. Given persistent circulatory compromise and the need for reliable vascular access, central venous catheterization was indicated.

A right femoral venous access was attempted by a senior anesthesiologist experienced in pediatric vascular access, using anatomical landmarks without ultrasound guidance. This represented the first and only puncture attempt. A 4‐Fr catheter was inserted using the Seldinger technique. Ultrasound guidance was not used initially as landmark‐guided femoral venous access was the routine practice in our setting at that time. No patient‐specific anatomical difficulties were identified, and the approach was performed in a standardized manner. The blood aspirated appeared venous in color, backflow was not under high pressure, and the guidewire advanced without resistance, all suggesting correct venous placement. However, during the advancement of the dilator, pulsatile arterial blood flow was unexpectedly observed, confirming inadvertent arterial puncture. The catheter and guidewire were immediately removed, and gentle manual compression was applied for 10 min.

Contralateral femoral central venous access was then successfully established under ultrasound guidance—a modality that was available in the operating room but had not been used for the initial attempt, given prevailing institutional practice at the time. A continuous infusion of norepinephrine was started, resulting in hemodynamic stabilization.

After stabilization, fracture reduction and pin fixation were performed (surgical duration approximately 45 min, no intraoperative complications).

Immediately following emergence from anesthesia, cyanosis of the right lower limb was noted (Figure 1), associated with prolonged capillary refill time, absence of distal pulses, decreased right femoral pulse compared with the contralateral side, equinus positioning, and monoparesis, with preserved sensory function.

**FIGURE 1:**
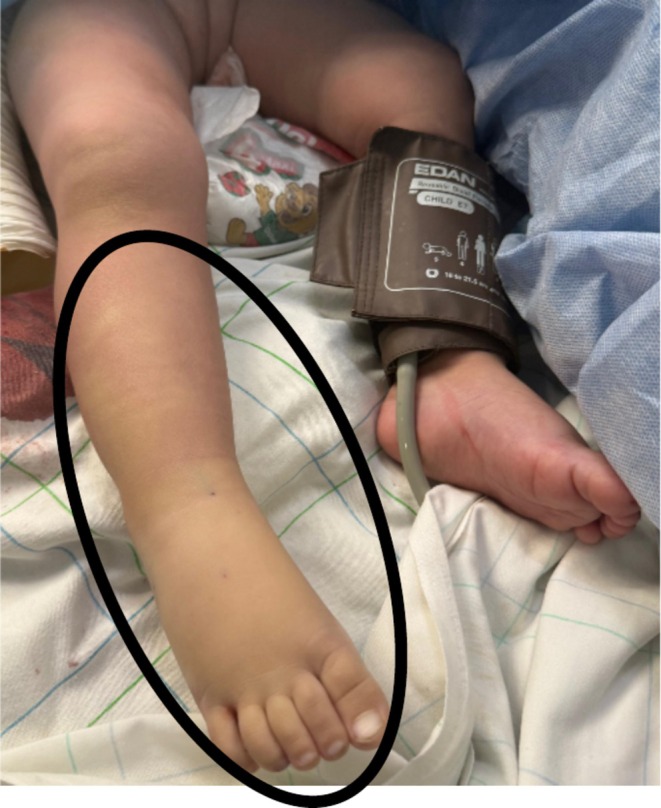
Image showing cyanosis of the right lower limb, equinus positioning, and the difference compared with the contralateral limb.

## 3 Management

The vascular surgery team was urgently consulted. Computed tomography angiography and Doppler ultrasound of the right lower limb confirmed occlusion of the right common femoral artery with reduced distal flow (Figure 2).

**FIGURE 2:**
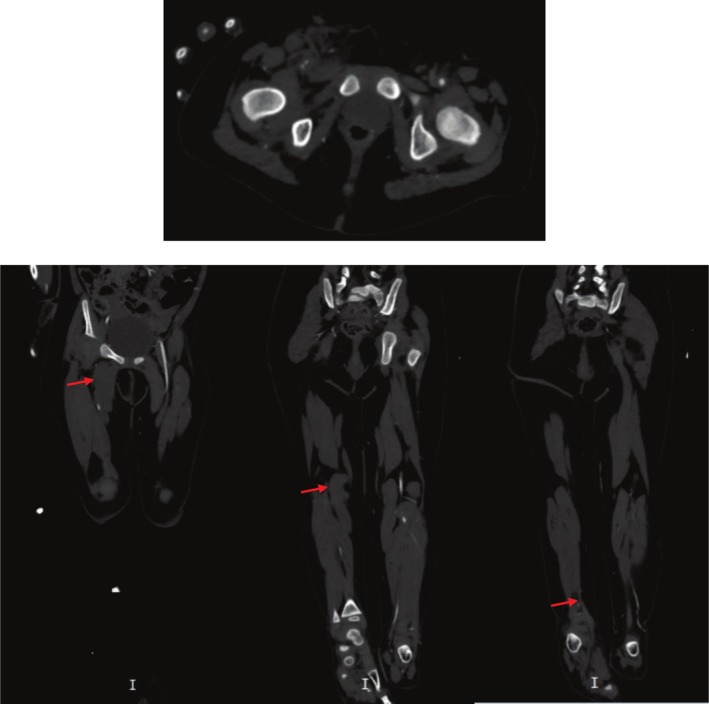
CT angiography of the right lower limb. (A–D) Representative axial and coronal images demonstrating interruption of arterial flow along the arterial axis of the right lower limb.

An intravenous bolus of unfractionated heparin (75 IU/kg) was administered prior to surgical intervention. Emergency thrombectomy via a right femoral triangle approach was performed. Intraoperatively, the puncture site of the common femoral artery was identified and a fresh thrombus was retrieved (Figure 3).

**FIGURE 3:**
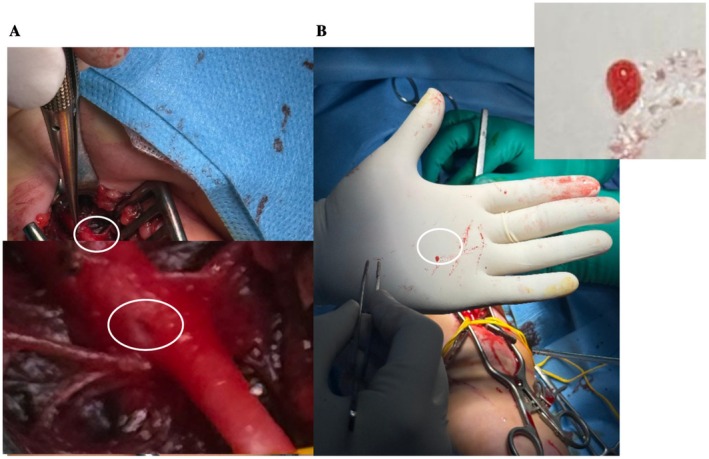
(A and B). Intraoperative findings: On the left (A), a lesion of the femoral artery; on the right (B), an arterial thrombus identified.

## 4 Outcome

Postoperative recovery was marked by restoration of satisfactory pulses in the right lower limb, with normal capillary refill time. Adequate blood flow was confirmed throughout all arterial segments on Doppler ultrasound (Figure 4). The patient was discharged on low‐molecular‐weight heparin (LMWH) therapy for three weeks and followed up at postoperative day 5 and day 21 with no complications.

**FIGURE 4:**
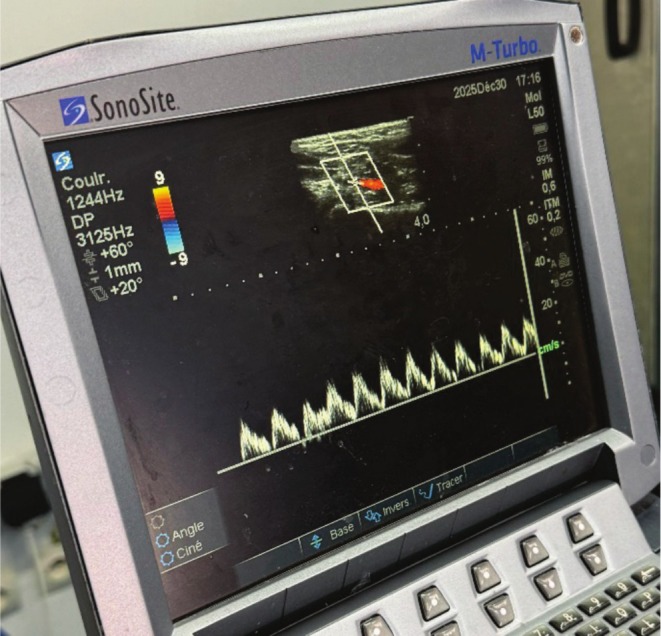
Postoperative spectral Doppler ultrasound of the right femoral artery demonstrating restoration of arterial flow following emergency thrombectomy.

## 5 Discussion

Femoral arterial injuries following central venous access in children are uncommon but well‐recognized complications, most often described in the context of arterial catheterization for cardiac interventions. Cases of acute limb ischemia arising after *venous* access attempts are far rarer. Flanigan et al. reported a series of iatrogenic pediatric vascular injuries in which the femoral artery was the most frequently injured vessel, though these were predominantly associated with arterial rather than venous procedures [1]. Bansal et al. [2] described femoral artery thrombosis after cardiac catheterization, with loss of distal pulses occurring in 2%–3% of cases. In contrast, our case is notable because ischemia developed after a single landmark‐guided femoral venous puncture—not an arterial procedure—and despite an experienced operator with initially reassuring procedural indicators. This closely parallels a small number of reported cases where venous access attempts have inadvertently resulted in arterial injury sufficient to cause acute limb ischemia, underscoring that the venous route does not confer immunity from such complications.

Several mechanisms likely converged in our patient to produce the severity of ischemia observed. The primary event was iatrogenic arterial injury at the femoral puncture site, confirmed intraoperatively by direct visualization of the vessel lesion and retrieval of a fresh thrombus. However, the relatively limited size of the thrombus retrieved did not appear commensurate with the degree of ischemic compromise, raising the hypothesis that arterial vasospasm made an additional contribution. It is well established that pediatric arteries—particularly in infants—are susceptible to vasospastic reactions following mechanical trauma or intraluminal instrumentation [3]. Importantly, vasospasm could not be directly demonstrated in our case, and this mechanism remains presumptive rather than proven; we acknowledge this as a hypothesis rather than a definitive finding.

Perioperative hemodynamic instability and the use of norepinephrine may also have contributed to distal hypoperfusion. Vasopressor‐induced acral ischemia and necrosis have been described in critically ill patients receiving catecholamine support, where intense vasoconstriction, endothelial dysfunction, and microcirculatory impairment can cause severe distal ischemia despite preserved large‐vessel patency [4]. In our patient, however, the clinical presentation and imaging findings were more consistent with acute arterial occlusion secondary to direct traumatic injury than with isolated vasopressor‐induced ischemia. The temporal relationship—ischemia appearing immediately after the procedure rather than after prolonged vasopressor exposure—supports this interpretation. Nonetheless, vasopressor use may have amplified the ischemic injury by impairing collateral perfusion.

Had revascularization been delayed or withheld, progression to irreversible limb ischemia would have been a serious risk. Severe ischemia may progress to dry gangrene and, in selected clinical contexts such as non‐operative management in high‐risk patients, to spontaneous autoamputation—a process in which prolonged ischemia leads to mummification and spontaneous separation of nonviable tissue along a demarcation line. Although primarily described in chronic ischemia, diabetic vasculopathy, frostbite, and vasopressor‐induced ischemia, autoamputation represents an extreme consequence of unmitigated tissue hypoperfusion and serves as a sobering reminder of the importance of early intervention [5]. In our case, prompt diagnosis and emergency thrombectomy within hours of ischemia onset enabled complete limb salvage.

Management of suspected iatrogenic arterial injury involves immediate removal of any indwelling catheter, administration of intravenous heparin unless contraindicated, and urgent vascular surgery consultation [1, 6]. Most patients with arterial vasospasm resolve with conservative management; however, 1%–10% ultimately require surgical revascularization [2, 7, 8]. Surgical options include thrombectomy, arterial repair, patch angioplasty, or bypass, with outcomes generally poorer in children younger than two years [7]. Delayed intervention may result in limb length discrepancy and long‐term functional impairment [1, 6]. Thrombolysis has been described, but carries significant bleeding risk (major complications in 40%–60% of reported cases) [7, 8].

The absence of ultrasound guidance during the initial access attempt likely played a significant role in this complication. In infants, the femoral vein and artery are in particularly close apposition, and vessel overlap has been reported in up to 12% of children aged 0–9 years [3]. Real‐time ultrasound guidance allows direct visualization of needle trajectory relative to vascular structures, reducing the likelihood of arterial puncture. The use of ultrasound for the contralateral placement in this case illustrates its practical feasibility even under emergency conditions. The use of ultrasound for vascular access in children is now a well‐established recommendation; this case reinforces adherence to that practice rather than proposing something novel. We acknowledge that in resource‐limited or emergency settings, landmark techniques may still be employed, but such use should be accompanied by heightened awareness of the risk of arterial injury and careful post‐procedural monitoring.

What makes this case particularly instructive is the convergence of features that individually might suggest a low‐risk scenario: a single puncture attempt, an experienced operator, no apparent anatomical difficulty, and initially misleading procedural indicators suggesting correct venous placement. Despite all of these reassuring elements, severe acute limb ischemia developed rapidly. This demonstrates that no individual risk factor in isolation can be relied upon to exclude arterial injury, and that even a single access attempt in an infant can result in limb‐threatening ischemia. It reinforces the importance of a high index of suspicion in all pediatric patients after femoral venous access, and the need for systematic postoperative neurovascular assessment.

## 6 Conclusion

This case illustrates that severe acute limb ischemia can develop following a single landmark‐guided femoral venous access attempt in an infant, even when performed by an experienced operator and without overt technical difficulty. Multiple mechanisms—arterial trauma with thrombosis, possible vasospasm, and hemodynamic compromise including vasopressor use—may compound the ischemic insult. Early clinical recognition, prompt vascular surgical intervention, and routine use of ultrasound guidance are essential.

## Author Contributions

**Anas El Hanbali:** conceptualization, data curation, investigation, writing – original draft. **Hicham Ziani:** project administration, supervision, validation. **Hamza Talbi:** data curation, writing – review and editing. **Leila Drouzi:** data curation, resources, writing – review and editing. **Mahmoud Byadi:** formal analysis, resources. **Zakariya Hamdani:** data curation, investigation, writing – review and editing. **Noussaiba Nabil:** supervision, validation. **Meryem Ennafiri:** writing – review and editing. **Saad El Harrak:** supervision, validation, writing – review and editing. **Larbi Ed‐Dafali:** methodology, validation, writing – review and editing. **Aziza Bentalha:** supervision. **Alae Koraichi:** supervision, validation. **Salma Ech Echerif El Kettani:** supervision, validation.

## Funding

The authors have nothing to report.

## Consent

Written informed consent was obtained from the patient's parents/legal guardians for publication of this case report and accompanying images. All data were anonymized.

## Conflicts of Interest

The authors declare no conflicts of interest.

## Data Availability

Data sharing is not applicable to this article as no datasets were generated or analyzed beyond those included in the report.
